# Dysphagia in a Patient With Lewy Body Dementia and Incidental Anterior Cervical Osteophyte: A Case Report

**DOI:** 10.7759/cureus.52968

**Published:** 2024-01-25

**Authors:** Gurjot Singh, Didar Singh, Gurleen Kaur, Piyush Puri, Tanya Ratnani, Ridhi Bhagat

**Affiliations:** 1 Internal Medicine, Saint John Regional Hospital, Springfield, USA; 2 Internal Medicine, Memorial Medical Center, Southern Illinois University, Springfield, USA; 3 Internal Medicine, Christian Medical College, Ludhiana, IND; 4 Internal Medicine, Adesh Institute of Medical Science and Research, Bathinda, IND; 5 Internal Medicine, Government Medical College, Bilaspur, Bilaspur, IND; 6 Internal Medicine, Teerthanker Mahaveer Medical College & Research Centre, Moradabad, IND

**Keywords:** anterior cervical osteophytes, lewy body dementia, alzheimer's disease, subacute dementia, rapid dementia, dementia with lewy body, dementia

## Abstract

One of the common problems affecting the elderly is dysphagia, which can be brought on by several things, including the presence of anterior cervical osteophytes. In this case study, a patient with Lewy body dementia is shown to have a case of dysphagia. The patient's primary complaint was dysphagia, which prompted questions regarding the development of underlying Lewy body dementia combined with gradual cognitive deterioration and motor control problems. An upper GI endoscopy was conducted during the patient's hospitalization after a barium swallow suggested esophageal obstruction but found no internal obstruction or any other abnormalities. Following the endoscopic procedure, the patient complained of neck aches. An anterior cervical osteophyte was subsequently discovered by computed tomography, which may have been the primary cause of the patient's dysphagia. The importance of considering coexisting medical conditions in elderly individuals, as well as the significance of promptly assessing and diagnosing dysphagia in the presence of neurodegenerative disorders such as Lewy body dementia, is emphasized by this example.

## Introduction

More than 75% of people aged 65 years and older have degenerative abnormalities in their cervical spine, including the presence of hypertrophic anterior cervical osteophytes. Osteophytes can be caused by various factors, including diffuse idiopathic skeletal hyperostosis, ankylosing spondylitis, degenerative processes, and previous cervical trauma with surgical interventions [1-3]. Although anterior cervical osteophytes are mostly asymptomatic, they can occasionally cause crippling symptoms, including dysphagia, dysphonia, and dyspnea [3-5]. The severity of these symptoms is frequently correlated with the size of osteophytic spurs. A 79-year-old man with Lewy body dementia was the subject of this case report, which included a case study of his presentation with dysphagia caused by a massive anterior cervical osteophyte. The patient chose not to undergo osteophytectomy; thus, his current conservative treatment consists of anti-inflammatory drugs and diet changes.

## Case presentation

A 79-year-old man with Lewy body dementia presented with difficulty in swallowing. It initially started eight months back but he was able to swallow solid food. As time passed, he had difficulty swallowing solid food and had shown signs of dysphagia for the past couple of weeks. He also showed signs of cognitive decline and difficulty with motor control. His dysphagia was progressive, and he had trouble swallowing solid foods and preferred to eat puree and liquid. He also showed signs of odynophagia but not dyspnea or dysphonia. He had a regular workup, and we performed a modified swallowing study to see how he swallowed. We observed an obstruction in the top third of the esophagus. We performed an upper GI endoscopy to determine if there were any internal obstructions, but it did not show anything.

**Figure 1:**
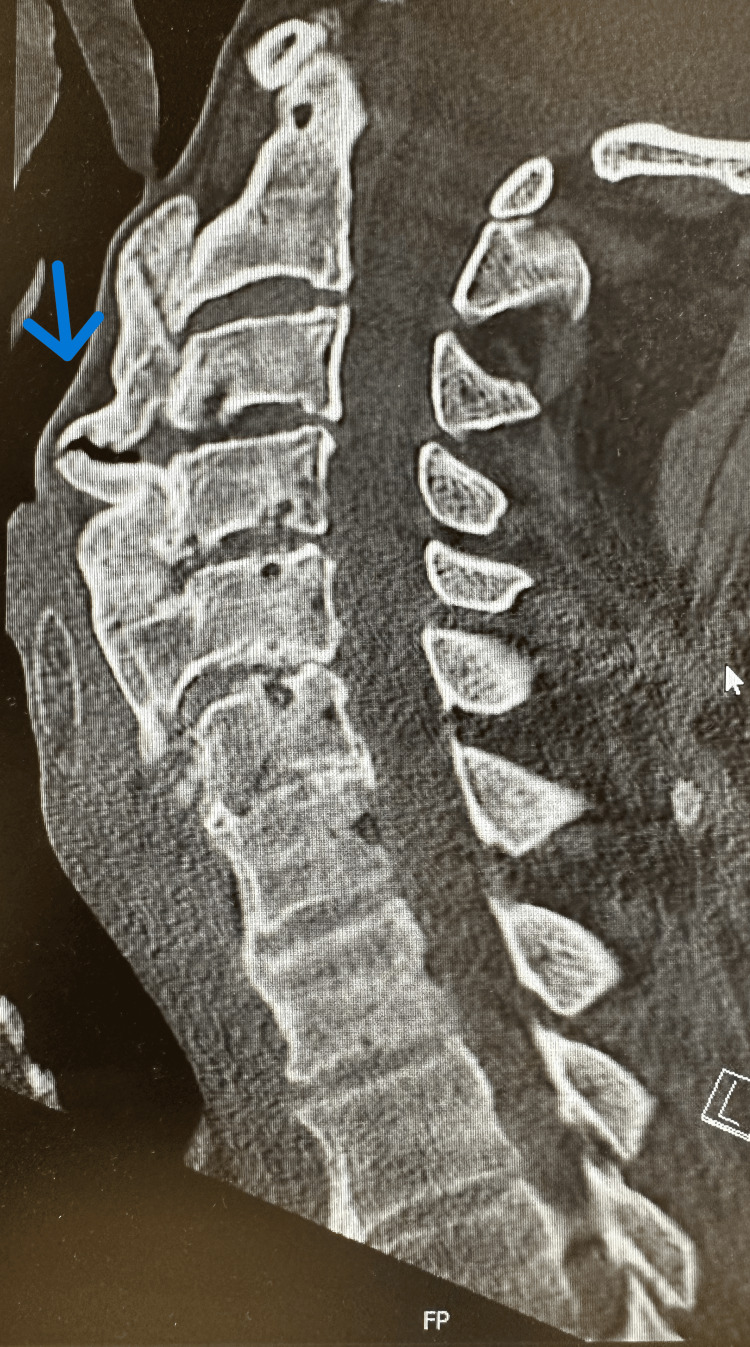
Largest osteophyte measuring 21 mm.

After the endoscopy, the patient complained of neck pain that was initially attributed to the procedure. However, the pain persisted for two days, prompting further investigation. Computed tomography (CT) of the cervical vertebrae showed significant anterior osteophytes extending from C2 to C6 (Figure 1).

The largest osteophyte was 21 mm in the anteroposterior dimension and impinged on the esophagus. The patient was eligible for conservative and surgical management. He decided to undergo conservative management, which included dietary adjustments and non-steroidal anti-inflammatory drugs (NSAIDs).

## Discussion

In elderly patients, dysphagia can be caused by various factors, and the onset of Lewy body dementia can worsen swallowing issues [6]. In this case, it is thought that the anterior cervical osteophytes may have worsened the dysphagia, resulting in the patient's main complaint. It is important to take an in-depth approach and suspect different causes of dysphagia in patients with a known neurodegenerative disorder, such as Lewy body dementia. Due to the cause of dysphagia and the patient's underlying Lewy body dementia, a multidisciplinary approach was adopted. This involved speech-language therapy, neurology, and gastroenterology to develop a treatment plan. Initially, a combination of diet modifications, swallowing therapy, and a conservative approach was used. However, if dysphagia persists and is debilitating with no improvement using the conservative approach, then, surgical intervention is necessary to remove the anterior cervical osteophyte.

The hypothesis regarding the origin of dysphagia due to hypertrophic cervical osteophytes is that it arises from direct compression of the aerodigestive tract and associated nerves, as well as local inflammation that leads to mucosal edema, adhesion formation, fibrosis, and cricopharyngeus muscle spasms. This local inflammation is believed to be caused by repetitive mechanical trauma resulting from the continuously moving pharyngolaryngoesophageal complex over the large hypertrophic hyperostosis. Osteophytes that usually cause dysphagia are located in the C5 cervical interspace [7].

MRI is typically performed in individuals who experience severe symptoms, including difficulty breathing and speaking. It is also performed in patients diagnosed with anterior cervical osteophytes preoperatively to assess any stenosis that can be addressed during the surgical procedure [8].

Surgery is often regarded as the cornerstone of treatment for many conditions by some surgeons. In particular, these medical professionals argue that cervical osteophytectomy should be a primary consideration for all cases of dysphagia resulting from a cervical osteophyte. By doing so, they believe that they can prevent the condition from worsening and potentially leading to acute respiratory distress [8]. Maiuri et al. provided evidence to support this viewpoint through a case report of a patient who experienced sudden respiratory distress and required an emergency tracheostomy due to chronic dysphagia caused by a cervical osteophyte [5].

## Conclusions

This case report underscores the importance of a comprehensive approach to diagnosing and managing dysphagia in elderly individuals with neurodegenerative disorders. The present case involved a patient with Lewy body dementia who experienced dysphagia that was ultimately attributed to an anterior cervical osteophyte. This report highlights the challenges in diagnosis, the necessity of multidisciplinary collaboration, and the importance of individualized treatment plans. The patient opted for conservative management, highlighting the significance of patient preferences in the decision-making process. Overall, this report contributes to our understanding of the less common causes of dysphagia in the elderly and emphasizes the need for ongoing research in this complex medical landscape.
